# Intraoral Approach: A Newer Technique for Filler Injection

**DOI:** 10.4103/0974-2077.63283

**Published:** 2010

**Authors:** Chytra V Anand

**Affiliations:** *Kosmoderma Clinic, Bangalore, India*

**Keywords:** Fillers, injection, intraoral

## Abstract

Filler injections are the most common aesthetic procedures used for volume correction. Various techniques have been described in the use of fillers. This article reviews the available literature on a new technique using the intraoral approach for injection of fillers.

## INTRODUCTION

A filler injection is one of the most frequently performed procedures in Aesthetic Dermatology practice. A dermatologist's goal is to achieve the best results with minimal or no downtime and the filler injection fulfils this goal satisfactorily. Filler injections are therefore an integral part of every aesthetic dermatologist's practice, be it in the form of temporary, semi-permanent, or permanent fillers. Different techniques of filler administration that are in common practice are linear threading (antegrade or retrograde), serial puncture, fanning, cross-hatching, depot, fern and cone.[1] These techniques use a percutaneous approach for injecting the filler. The choice of technique depends on the site of injection, the product and the result desired. This article reviews a new technique, an intraoral approach, to place the fillers in the injection plane in the mid-face rejuvenation and perioral region. This procedure is recommended for heavier molecule products like Restylane Sub Q or Juvederm Voluma and has been introduced to achieve minimum downtime.[2]

## TECHNIQUE FOR MID-FACE AUGMENTATION USING THE INTRAORAL ROUTE

The skin overlying the treatment area is cleansed with a topical antiseptic. With the patient in an upright position, the procedure area is marked. The areas are marked using the Hinderers technique.[3] The volume augmentation site is identified by two lines intersecting each other. One line runs from the tragus to the alar cartilage of the nose and the other from the outer canthus of the eye to the labial commissure. The implant is placed in the upper outer quadrant of the criss-cross lines.

The oral cavity is cleaned with Betadine™ mouth gargle or a chlorhexidine-based mouth wash, and a local anaesthetic infiltration of xylocaine 2% with adrenaline (1:2,00,000) is given at the mucosal puncture sites in the upper gingival fornix at the second incisor and canine junction level. After the lips are retracted by an assistant, a small stab or puncture wound is made in the mucosa using a scalpel blade No. 11. A blunt 18 gauge cannula is introduced through this site superficial to the bone, always using the other hand to guide the cannula. The guiding hand is kept at the infraorbital margin to prevent placing of the product in the orbital fossa. Multiple tracts are made with the cannula and the filler is placed in retrograde technique till the desired volume is reached. A fresh cannula is used for the other side of the face. An antibiotic prophylaxis is used to combat the risk of infection.

## ADVANTAGES OF INTRAORAL FILLER ADMINISTRATION

1. As Aesthetic Dermatologists usually use the intraoral approach for performing blocks and for performing chin implants, this technique of the intraoral approach is familiar to most aesthetic dermatologists. It is claimed therefore that no separate training is needed.
2. It has been reported that intraoral administration of fillers can reduce the swelling in the plane of injection. This can lead to less distortion during the procedure and thereby a more accurate assessment and placement of the product at the site.
3. The transoral injections produce less soft tissue trauma with minimal erythema and edema in the postoperative period.[4] Post procedure sequelae of edema and bruising is lessened, and hence, there is an additional benefit of lessened downtime for the patient.
4. This method allows easy access to the zygomatic periosteum, thus decreasing the chances of disruption of fascia, muscles, nerves and blood vessels in the area.
5. It is also felt by some doctors that the salivary enzymes such as lysozymes, peroxidases are bacteriostatic or bacteriocidal, and can therefore act as the first line of defence in preventing infection.[5,6]

## DISADVANTAGES

As with any new technique, several apprehensions have been expressed about this technique too, which are as follows:

1. Risk of infection: The oral cavity has plenty of resident bacteria, and injection through this route has the potential risk of introducing infection into the procedural site.[2]
2. Intraoral approach requires advanced skill, proper anatomic knowledge and experience, as it is a more difficult approach than the transcutaneous method. It requires exact precision to avoid injecting directly around the mental and temporal nerves. Also one of the key blood vessels to the infraorbital periorbital fat is located right in the middle of the injection path. All these make this a tougher approach.

A recent report has claimed that pre-mixing fillers with lidocaine can lead to less bruising and swelling after transcutaneous administration.[7] In view of this, intraoral administration is no longer needed to obtain such an advantage.

## CONCLUSIONS

In view of these features, it can be concluded that the claimed benefits of the intraoral approach are weighed by the risks and difficulties of this technique, and there is no added advantage of the intraoral approach over the traditional transcutaneous route, unless one is using a heavier molecule filler for volume augmentation.
